# Progress in the Mechanism of the Effect of Fe_3_O_4_ Nanomaterials on Ferroptosis in Tumor Cells

**DOI:** 10.3390/molecules28114562

**Published:** 2023-06-05

**Authors:** Yaxuan Wang, Xiao Wu, Xiaoying Bao, Xianbo Mou

**Affiliations:** 1 Health Science Center, Ningbo University, Ningbo 315211, China; wangyaxuan0718@163.com (Y.W.); juanjuanmiemie@163.com (X.B.); 2 The First Affiliated Hospital of Ningbo University, Ningbo 315211, China; 3 Key Laboratory of Early Prevention and Treatment for Regional High Frequency Tumor, Ministry of Education, Nanning 530021, China; 4 Guangxi Key Laboratory of Early Prevention and Treatment for Regional High Frequency Tumor, Guangxi Medical University, Nanning 530021, China

**Keywords:** Fe_3_O_4_ nanoparticles, ferroptosis, ROS, tumors

## Abstract

Ferroptosis is a new form of iron-dependent programmed cell death discovered in recent years, which is caused by the accumulation of lipid peroxidation (LPO) and reactive oxygen species (ROS). Recent studies have shown that cellular ferroptosis is closely related to tumor progression, and the induction of ferroptosis is a new means to inhibit tumor growth. Biocompatible Fe_3_O_4_ nanoparticles (Fe_3_O_4_-NPs), rich in Fe^2+^ and Fe^3+^, act as a supplier of iron ions, which not only promote ROS production but also participate in iron metabolism, thus affecting cellular ferroptosis. In addition, Fe_3_O_4_-NPs combine with other techniques such as photodynamic therapy (PDT); heat stress and sonodynamic therapy (SDT) can further induce cellular ferroptosis effects, which then enhance the antitumor effects. In this paper, we present the research progress and the mechanism of Fe_3_O_4_-NPs to induce ferroptosis in tumor cells from the perspective of related genes and chemotherapeutic drugs, as well as PDT, heat stress, and SDT techniques.

## 1. Introduction

Global Statistics of 2020 [1] showed that there were an estimated 19.3 million new cancer cases and nearly 10 million cancer deaths, of which female breast cancer surpassed lung cancer as the most commonly diagnosed cancer with an estimated 2.3 million new cases (11.7%), followed closely by lung cancer (11.4%), colorectal cancer (10.0%), prostate cancer (7.3%), and stomach cancer (5.6%). Lung cancer remained the leading cause of cancer deaths with an estimated 1.8 million deaths (18%), followed by colorectal cancer (9.4%), liver cancer (8.3%), stomach cancer (7.7%), and female breast cancer (6.9%). The proliferation and apoptosis of corresponding tumor cells are closely related to tumor formation and progression. Thus, in the past few decades in oncology, scientific researchers have been devoted to the development of various apoptosis-related drugs to reverse the fate of non-senescent and over-proliferating tumor cells [2,3,4].

However, the therapeutic outcome is far from satisfactory due to the inherent or acquired apoptosis resistance of tumor cells [5]. Lung cancer, with the highest age-standardized incidence rate (ASIR) and age-standardized mortality rate (ASMR) worldwide, and with 2.1 million new cases and 1.8 million deaths every year, has a very high mortality rate [6,7,8,9]. Small cell lung cancer (SCLC) is one of the most malignant types of lung cancer. It has a short multiplication time, patients are prone to rapid drug resistance during treatment, and the disease deteriorates rapidly after recurrence. Currently, there is no effective second-line single-agent chemotherapy regimen other than topotecan [10]. In addition, the incidence and mortality of hepatocellular carcinoma (HCC) has rapidly increased worldwide [11]. HCC is an aggressive and highly malignant liver tumor with poor survival rates, and most patients are clinically diagnosed at an advanced stage [12]. The pathogenesis of the tumor has been of great interest to researchers; however, due to its diversity and complexity, it still has a long way to go despite the large amount of human and material resources invested every year [13,14,15,16,17]. In recent years, a new form of cell death different from the apoptotic mechanism, namely ferroptosis, has attracted attention.

Ferroptosis, first proposed in 2012 by Scott J. Dixon et al., is a unique form of iron-dependent non-apoptotic cell death triggered by the oncogenic RAS-selective lethal small molecule Erastin [18]. Early studies found that cysteine is required for the growth and death inhibition of cells, and the absence of cystine in the culture medium resulted in the death of human fibroblasts due to glutathione (GSH) depletion. Extracellular and intracellular cysteine are required to maintain GSH biosynthesis and inhibit mammalian cell death, which could be prevented and treated with the lipophilic antioxidant (α-tocopherol) and iron chelators (deferoxamine) [19]. In the following years, with the further study of the mechanism of ferroptosis, it has gradually become a promising cancer treatment.

Compared with other metal nanomaterials [20,21,22,23], Fe_3_O_4_-NPs are one of the few FDA-approved nanomaterials for clinical use, which has simple preparation [24,25,26], safe and stable chemical properties, and good biocompatibility [27,28,29]. Moreover, it has been widely used in pathogen detection [30,31,32,33,34], tumor diagnosis and treatment [35,36,37], gene mutation analysis [38,39,40,41,42], targeted drug delivery [43,44,45], and nuclear magnetic resonance imaging (MRI) [46,47,48]. In addition, recent studies have shown that Fe_3_O_4_-NPs, due to their rich Fe^2+^ and Fe^3+^ contents, can affect the iron metabolism of tumor cells, increase local ROS levels in tumor tissues, participate in or even induce the occurrence of ferroptosis in tumor cells, and then be able to inhibit or kill tumor cells. In this paper, the research progress and mechanism of Fe_3_O_4_-NP-induced ferroptosis in tumor cells were reviewed.

## 2. Ferroptosis

Ferroptosis, a new form of cell death discovered in recent years, is an iron-dependent non-apoptotic form of cell death characterized by the accumulation of intracellular ROS [18]. This modality disrupts the redox homeostasis of cells and has a great potential to kill cancer cells [49,50]. Studies on the mechanisms of ferroptosis have focused on lipid peroxidation (LPO) and iron loading. Under normal circumstances, ROS is mainly produced by normal metabolism in mitochondria and is essential for cell signaling and tissue homeostasis [51]. Polyunsaturated fatty acid (PUFA) peroxidation induced by ROS is the main cause of ferroptosis. Furthermore, ferroptosis is regulated by a complex interaction between cellular redox homeostasis, lipid metabolism, and iron metabolism [52]. In terms of cell morphology, cells undergoing ferroptosis show some specific changes, such as the appearance of smaller mitochondria than normal, wrinkled mitochondrial membranes, reduced or absent mitochondrial cristae, and fragmented extracellular membrane [53]. The biochemical features of ferroptosis are cystine deficiency, GSH depletion, and glutathione peroxidase 4 (GPX4) inactivation [54]. However, the underlying mechanisms have not been fully elucidated.

**Figure 1:**
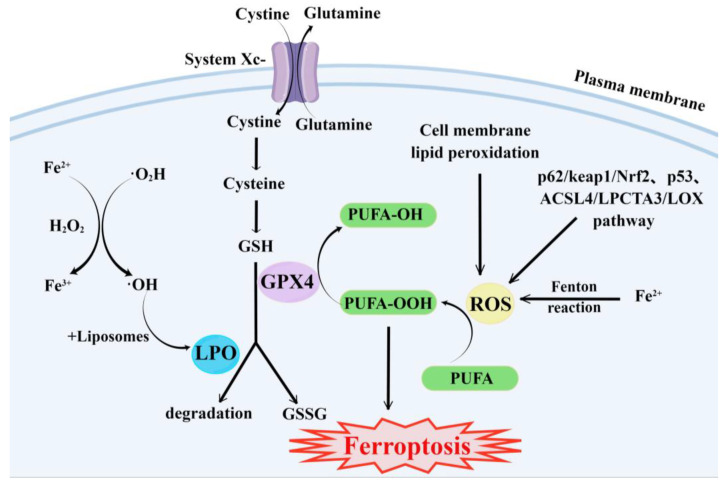
Major metabolic pathways of ferroptosis. By Figdraw.

The main cause of ferroptosis is the change of cell metabolism, which leads to the accumulation of LPO and ROS in the cells. According to existing research reports, the changes in cell metabolic pathways caused by ferroptosis mainly include the following aspects (Figure 1):

(1) GSH synthesis pathway is inhibited and LPO accumulates, thus inducing ferroptosis in cells [55].
(2) Iron metabolism is altered. Iron is a redox-active metal involved in ROS formation and LPO diffusion, and a rising iron level could increase cellular susceptibility to iron-dependent cell death [56]. The accepted explanation today is that Fe^2+^ can transfer electrons to intracellular oxygen, and then react with intracellular lipids to form LPO, which further induces ferroptosis [57,58]. Iron metabolism genes and iron metabolism regulation genes play a key role in intracellular system iron homeostasis. For example, the gene of Iron Responsive Element Binding Protein 2 (IREB2) is a key player in the Erastin-induced ferroptosis of HT-1080 fibrosarcoma cells and Calu-1 lung cancer cells [59]. Thus, intracellular iron overload is critical for ferroptosis [60].
(3) ROS metabolic pathway effects. This pathway also plays an important role in ferroptosis. Cytosolic cystine/glutamate transport receptor (System Xc-) and voltage-dependent anion channels (VDACs) [18] in the outer mitochondrial membrane, GPX4 and ferroptosis suppressor protein 1 (FSP1) ferroptosis-related proteins [61,62], and p62/keap1/Nrf2 [63], p53-related pathway [64,65], and ACSL4/LPCTA3/LOX [66] ferroptosis-related pathways play their roles in regulating ferroptosis by affecting ROS metabolism pathways [67].

## 3. Mechanism of the Effect of Fe_3_O_4_-NPs on Ferroptosis in Tumor Cells

Research on tumor detection, diagnosis, and treatment methods has been a popular topic, and the development of nanotechnology has provided new strategies in this field [68,69,70,71,72,73,74,75]. Fe^2+^ and Fe^3+^, rich in Fe_3_O_4_-NPs, contain abundant lone-pair electrons and can form coordination bonding with organic molecules. Thus, Fe_3_O_4_-NPs can easily realize surface modification and surface drug loading. After surface modification, Fe_3_O_4_-NPs are more difficultly encapsulated by plasma proteins and can avoid the phagocytosis of the reticuloendothelial system, reduce their clearance rate, and prolong the circulation time in blood [76,77,78,79].

**Figure 2:**
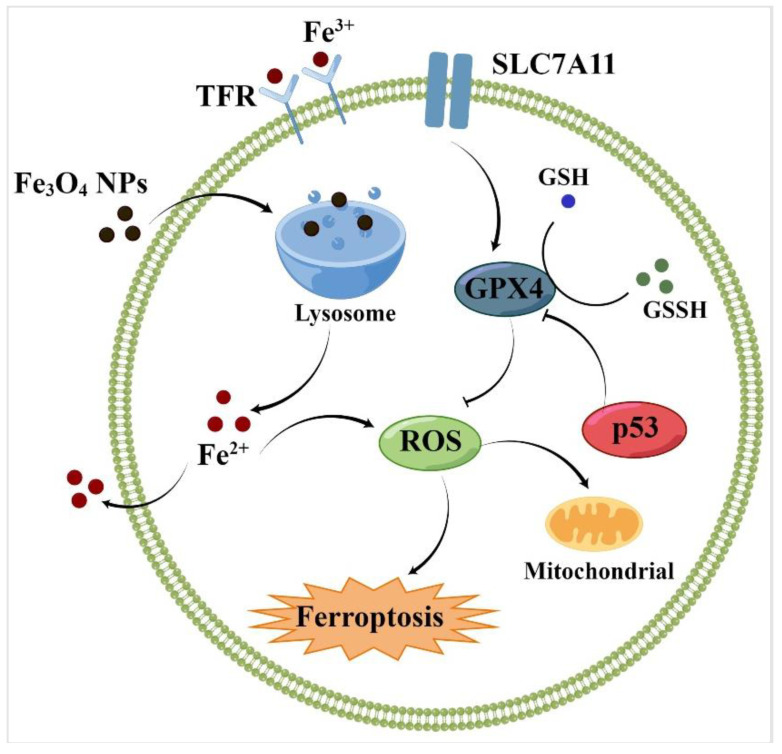
Effect of Fe_3_O_4_-NPs on cell ferroptosis. By Figdraw.

Moreover, both Fe^2+^ and Fe^3+^ can react with hydrogen peroxide to generate ROS, catalyze the occurrence of the Fenton reaction, and then induce ferroptosis in cells [57]. Furthermore, it can enhance immunotherapy, which has attracted much more attention. Overall, iron-based nanoparticles as an emerging ferroptosis inducer can enhance iron ion and ROS levels. For example, Fe_3_O_4_-based nanoparticles prepared by Song et al. can generate ROS, induce intracellular oxidative stress, and accelerate the Fenton reaction to increase the local concentration of Fe^2+^, Fe^3+^, and H_2_O_2_ to kill cancer cells (Figure 2) [80].

**Figure 3:**
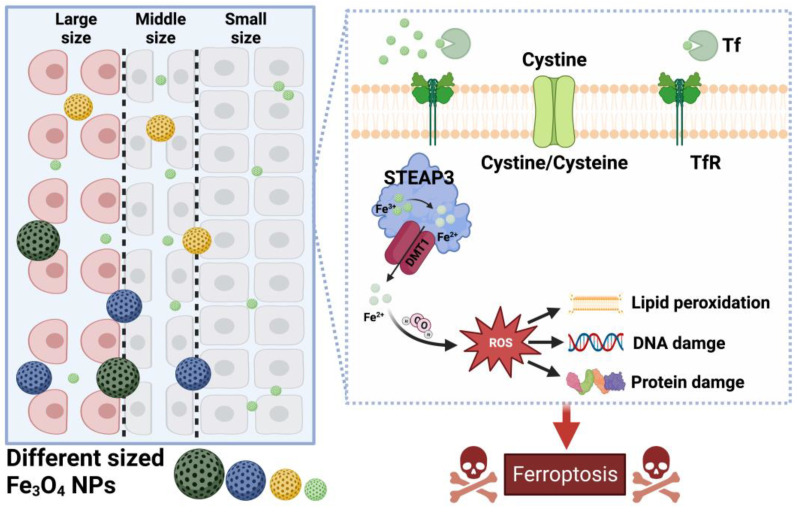
Effect of particle size parameters of Fe_3_O_4_-NPs on cell ferroptosis. By Figdraw.

Tian et al. [81] synthesized Fe_3_O_4_-NPs with different sizes (with an average size of 2, 4, 10, and 100 nm) and evaluated their antitumor effects (Figure 3). The experimental results showed that, compared to larger Fe_3_O_4_-NPs, the ultra-small (<5 nm) Fe_3_O_4_-NPs could accumulate in the nucleus and had a higher efficiency of •OH and ROS generation and a higher ability of cytokines secretion. Additionally, the ultra-small Fe_3_O_4_-NPs were more efficient in enhancing the toxicity of H_2_O_2_ due to the rapid release of Fe^2+^. Nevertheless, 10 nm Fe_3_O_4_-NPs displayed the best antitumor effect in vivo. It shows that selecting the right size of Fe_3_O_4_-NPs is more effective in treating tumors.

Ferulic acid glycerol, a nano-Fe supplementation, was found to have antileukemic effects under low levels of iron transport protein (FPN) in leukemic cells by inducing ferroptosis in vitro and in vivo [82]. Leukemic cells are unable to export intracellular Fe^2+^ produced by methyl ferulate, which can cause an increase in ROS levels through the Fenton reaction and then affect leukemic cells [83]. In addition, Zanganeh et al. showed that triglyceride ferulate has an intrinsic therapeutic effect on the growth of liver and lung metastases in early breast cancer and lung cancer [84].

### 3.1. Effect of Fe_3_O_4_-NPs on the Expression of Ferroptosis-Related Genes

**Figure 4:**
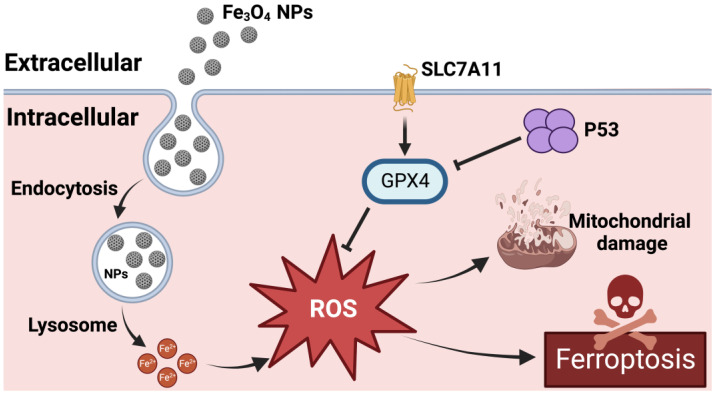
Mechanism of Fe_3_O_4_-NP-induced ferroptosis through up-regulation of p53. By Figdraw.

In addition to lipid peroxidation [85,86,87], the role of genes involved in ferroptosis should also not be ignored. It has been shown that p53 activation is required for ferroptosis in cancer cells [88]. p53 can mediate ferroptosis in fibroblasts, osteosarcoma cells, and breast cancer cells through the transrepression of SLC7A11 [64,88]. It is the first piece of evidence of ferroptosis-induced p53 gene expression. p53 plays a dual role in ferroptosis. On the one hand, p53, as a tumor suppressor, down-regulates SLC7A11, thereby inhibiting the cellular uptake of cysteine, increasing cellular LPO and ultimately leading to ferroptosis. On the other hand, p53 inhibited Erastin-induced ferroptosis by blocking dipeptidyl peptidase 4 (DPP4) activity in human colorectal cancer (CRC) [89]. Fe_3_O_4_-NPs could promote the expression of p53 and acetylated p53 protein and accelerate their translocation from cytoplasm to nucleus in human umbilical vein endothelial cells; silencing p53 could up-regulate SLC7A11 and GPX4 mRNA expression levels (Figure 4) [90]. For macrophages, RNA sequencing results showed that Fe_3_O_4_-NPs induced ferroptosis through up-regulating p53 gene expression and down-regulating SLC7A11 gene expression [91]. In ovarian cancer cells incubated with superparamagnetic iron oxide, iron repletion could promote p53 expression [92,93] and up-regulated p53 promoted ferroptosis. These results suggest that Fe_3_O_4_-NPs could promote the expression of p53, which is involved in the expression of SLC7A11 and GPX4 to regulate ferroptosis.

In addition, there are other genes that play an equally important role in ferroptosis such as BRCA1-Associated Protein 1 (BAP1). BAP1, as a tumor suppressor, promotes ferroptosis and inhibits cancer cell growth by inhibiting SLC7A11 [94]. The mechanism may be that BAP1 suppresses SLC7A11 transcription by reducing histone 2A ubiquitination (H2Aub) on the SLC7A11 gene. Gao et al. [83] developed a cancer treatment named gene interference ferroptosis therapy (GIFT) by combining a DMP-controlled gene interference tool with DMSA-coated Fe_3_O_4_-NPs. GIFT consists of a gene interference vector (GIV) and Fe_3_O_4_-NPs, and DMP, an NF-κB-specific promoter, consisting of an NF-κB decoy and a minimal promoter. In a variety of cancer cells treated with Fe_3_O_4_-NPs, the expression of FPN and LCN2 (iron metabolism genes) was selectively down-regulated by Cas13a or microRNA controlled by the NF-κB-specific promoter and ferroptosis was significantly induced, but the same treatment had little effect on normal cells.

The Beclin1/ATG5-dependent autophagy pathway is also closely related to ferroptosis induced by Fe_3_O_4_-NPs. Wen et al. [95] demonstrated that down-regulated Beclin1/ATG5 could significantly inhibit the ferroptosis induced by ultra-small iron oxide nanoparticles (USIONPs), while overexpressed Beclin1/ATG5 could significantly enhance ferroptosis. It suggests that USIONP-induced ferroptosis is regulated through the Beclin1/ATG5-dependent autophagy pathway, which could lead to the degradation of USIONPs, the release of iron ions, and the accumulation of ROS and LPO, and ultimately induce cellular ferroptosis.

### 3.2. Fe_3_O_4_-NPs Enhance the Sensitivity of Tumor Cells to Anticancer Drugs

**Figure 5:**
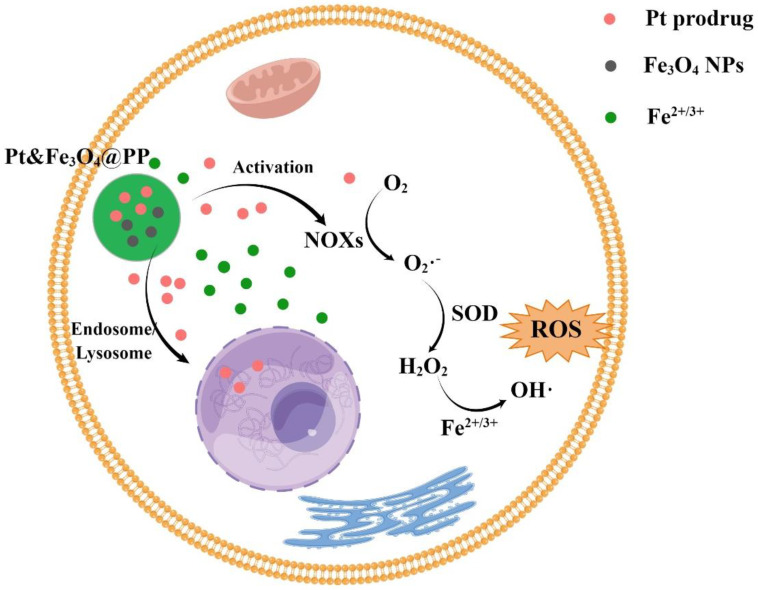
Mechanism of Pt&Fe_3_O_4_@PP-induced ferroptosis. By Figdraw.

There is growing evidence that the induction of ferroptosis enhances susceptibility to anticancer drugs [96,97]. Inducing ferroptosis with Erastin and RSL-3 has been reported to enhance the anticancer effects of cisplatin and reduce the resistance of anticancer drugs by inhibiting System Xc- in lung cancer, CRC, ovarian cancer, and pancreatic ductal adenocarcinoma [98]. Fe_3_O_4_-NPs provide new ideas for improving the sensitivity of tumors to chemotherapeutic drugs by inducing ferroptosis, while providing new methods for in vitro high-sensitive detection [17,68,99,100,101]. Gao et al. [102] prepared a peptide carrier (Pt&Fe_3_O_4_@PP) (Figure 5) encapsulated with the anticancer drug cisplatin (Pt drug) and Fe_3_O_4_-NPs. Cisplatin, as an inducer of ferroptosis and apoptosis for NSCLC A549 cells, plays an important role in the mechanism of ferroptosis through reducing GSH depletion and inducing GPX4 inactivation [103]. The tumor microenvironment (TME) triggers the release of Pt and Fe^2+^/Fe^3+^, which in turn induces an intracellular cascade reaction to produce sufficient ·OH for ferroptosis treatment. In addition, the release of Pt could induce apoptosis in tumor cells, and Fe_3_O_4_-NPs can be used for T2-weighted imaging of tumors. It was further found that the combination of cisplatin and class I FIN Erastin had a significant synergistic effect on its antitumor activity. Liu et al. [104] established a novel drug delivery system (Fe_3_O_4_-siPD-L1@M-BV2) targeting glioblastoma multiforme (GBM). This system could induce ferroptosis in drug-resistant GBM cells and matured dendritic cells (DC), increase the proportion of M1 and M2 microglia in drug-resistant GBM tissues, and then inhibit the in situ growth and prolong the survival time of drug-resistant GBM mice. PD-1/PD-L1 inhibitors could activate T cells to secrete γ-interferons [105]. These γ-interferons inhibit cysteine transporters, thereby preventing cysteine uptake and reducing glutathione synthesis in cancer cells, and finally enhancing ferroptosis. Both in combination with Fe_3_O_4_-NPs and with the drug itself, the drug systems have a facilitative effect on the onset of ferroptosis by reducing glutathione in cancer cells.

Drug-loaded microspheres, as a commonly used clinical chemoembolic agent, suffer from inhomogeneous particle size and unstable efficacy. Chen et al. [106] introduced Fe_3_O_4_-NPs into the chemoembolic agent system to prepare gelatin microspheres co-loaded with doxorubicin (ADM/Fe_3_O_4_-MS) to improve its anti-liver cancer effect. Ferroptosis was also involved in the process of tumor cell death, in which GPX4, a marker of ferroptosis, was significantly reduced and ACSL4 was significantly increased. Meanwhile, ferroptosis inhibitors reversed the killing effect induced by ADM/Fe_3_O_4_-MS plus microwave irradiation. This study demonstrates that Fe_3_O_4_-NPs can significantly improve the overall efficacy of drug-loaded microspheres, which has promising applications especially in tumor chemoembolization [107].

Ferroptosis plays an important role in the treatment of triple-negative breast cancer (TNBC) cells. Yao et al. [108] prepared a novel ferroptosis nanodrug by loading simvastatin (SIM) into amphoteric polymer-coated magnetic nanoparticles (Fe_3_O_4_@PCBMA) to improve the therapeutic effect of TNBC. SIM could inhibit the expression of HMG-CoA reductase (HMGCR) and down-regulated the mevalonate (MVA) and GPX4 pathways, and consequently induced ferroptosis in cancer cells. Fe_3_O_4_-NPs serve as a good carrier to deliver antitumor drugs to tumor tissues through passive targeting. Based on this study, it is known that an Fe_3_O_4_-NP nanosystem can overcome the drug resistance caused by apoptotic drugs in tumor cells in clinical practice.

**Figure 6:**
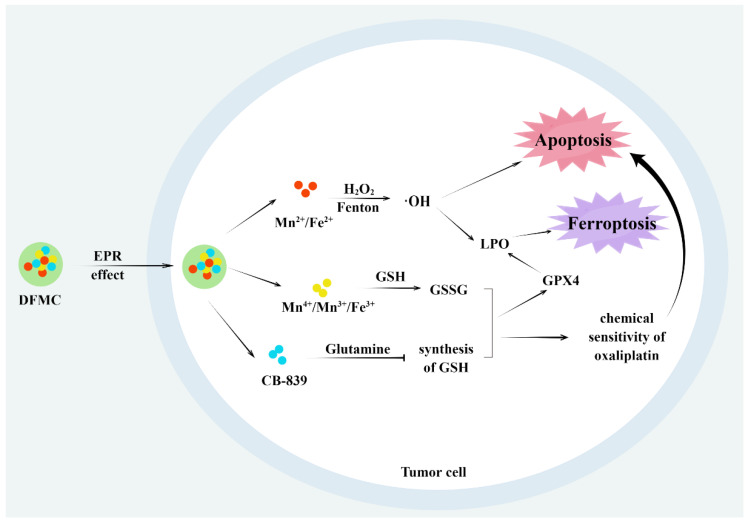
Mechanism of DFMC-induced ferroptosis. By Figdraw.

Wu et al. [109] developed a nanomedicine with both nanocatalytic and glutaminase inhibitor properties, which was made by integrating ultrasmall Fe_3_O_4_-NPs and CB-839 into dendritic mesoporous silica nanoparticles and then etched with Mn (DFMC) (Figure 6). The Fe^2+^ and Mn^2+^ in DFMC exerted Fenton/Fenton-like activity and could effectively catalyze the decomposition of H_2_O_2_ to be highly toxic ·OH under acidic conditions for tumor therapy. The GSH depletion ability of DFMC not only consumes existing GSH, but also prevents the synthesis of GSH, which weakens the GSH-related antioxidant defense system (ADS), thereby enhancing the sensitivity of ROS-mediated tumor catalytic therapy and oxaliplatin chemotherapy, and finally achieving higher tumor clearance rate.

### 3.3. Fe_3_O_4_-NPs Can Enhance the Efficacy of Drugs or Synergize with Them to Promote Ferroptosis

In one study, magnetic nanoparticles (MNP) were coated with the zwitterionic polymer Poly (2-methacryloyloxyethyl phosphorylcholine) (PMPC) and then loaded with sorafenib (SRF) to obtain the drug-loaded nanoparticles composite MNP@PMPC-SRF [110].

Fe_3_O_4_-NPs can also improve the anticancer efficiency of artemisinin (ART). The PFH/ART@PLGA/Fe_3_O_4_-EFA constructed by Wang et al. [110] was exposed to Low Intensity Focused Ultrasound (LIFU) irradiation to induce perfluorohexane (PFH) phase transition and nanoparticle collapse, which promoted the release of ART and Fe_3_O_4_ in vitro. The efficiency of this approach is attributed to the synergistic effect of intracellular Fe^2+^ and ART, which play a key role in the induction of ferroptosis in tumor cells by promoting ROS production in vitro.

## 4. Fe_3_O_4_-NPs in Combination with PDT, Heat Stress, and SDT Further Induced Ferroptosis in Tumor Cells

The combination of Fe_3_O_4_-NPs with other technologies further exploits their advantages and expands the scope of application [111,112,113,114,115,116]. The results of established studies have shown that Fe_3_O_4_-NPs are of great interest as a good cellular ferroptosis inducer, especially in the field of tumor therapy. However, current iron-based nanoparticles, due to their low ROS generation efficiency, require synergistic use with other therapeutic approaches or high dose application to achieve effective treatment [117].

### 4.1. Synergy of Photodynamic Therapy (PDT)

PDT is a novel tumor treatment method. However, the efficacy of PDT is greatly affected by oxygen concentration [118], which applied alone is not satisfactory. Fe_3_O_4_-NPs are highly biocompatible in vivo. When they enter cancer cells by endocytosis, they can be effectively released into Fe^2+^ and Fe^3+^ in the acidic environment of cancer cells, thus enhancing the Fenton reaction [119,120]. The Fenton reaction can produce ·OH and O_2_, increase the oxygen concentration in tumor cells, enhance the PDT effect, and then realize the treatment of cancers.

**Figure 7:**
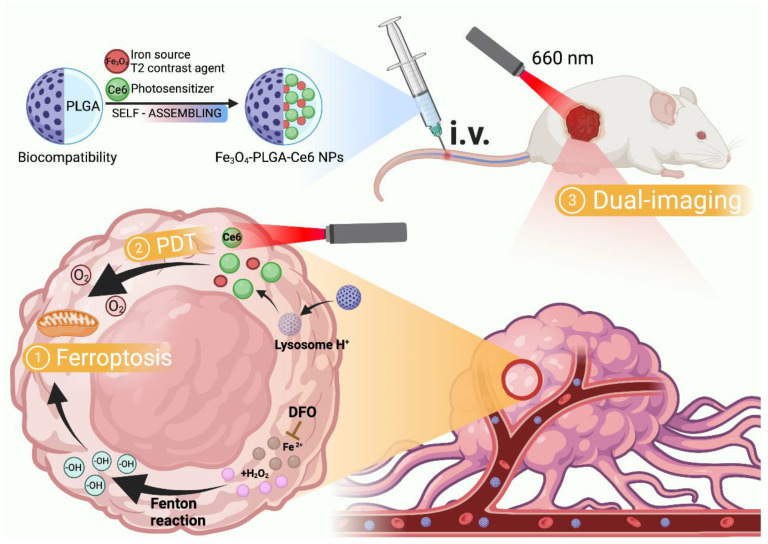
Mechanism of PLGA-induced ferroptosis. By Figdraw.

Chen et al. [121] designed a nanosystem coated with the FDA-approved poly (lactic-co-glycolic acid) (PLGA) containing Fe_3_O_4_-NPs and chlorin E6 (Ce6) for synergistic ferroptosis-photodynamic anticancer therapy (Figure 7). The nanosystem can dissociate in acidic TME to release Fe^2+^/Fe^3+^ and Ce6. Fe^2+^/Fe^3+^ may react with the excess intracellular H_2_O_2_ to trigger the Fenton reaction, which allows intracellular ROS accumulation and LPO generation, providing the necessary conditions for the onset of ferroptosis. Meanwhile, Ce6 could also increase the generation and accumulation of ROS under laser irradiation, and then provide PDT to further promote cellular ferroptosis. It demonstrates that the synergistic effect of PDT and ferroptosis induced by Fe_3_O_4_-NPs has an efficient effect on tumor suppression.

Ultrafine Fe_3_O_4_@PGL complex nanoparticles, designed by Liang et al. [122], were formed by amphiphilic porphyrin-grafted lipid (PGL) self-assembly on the hydrophobic surface of ultrasmall Fe_3_O_4_-NPs. This complex could not only trigger PDT under laser action, but also induce a large amount of ROS through the activity of tumor-associated macrophages (TAMs). Experimental results confirmed that most cells followed a PDT-based cell death pathway, and Fe_3_O_4_-mediated ferroptosis can further improve the therapeutic efficiency, showing a strong synergistic effect. Under the synergistic effect of ferroptosis and PDT, tumor cells regressed [123].

In summary, Fe_3_O_4_-NPs in an acidic environment (such as TME and lysosomes) can dissociate and then release Fe^2+^/Fe^3+^ diffusing into the cytoplasm, which accelerates the Fenton reaction, promotes ferroptosis, and leads to intracellular ROS accumulation and LPO generation [124]. PDT can also promote ROS production and accumulation, which in turn promotes the occurrence of ferroptosis. Therefore, the combination of Fe_3_O_4_-NPs and PDT can effectively promote ferroptosis.

### 4.2. Metabolism Modulation by Heat Stress

**Figure 8:**
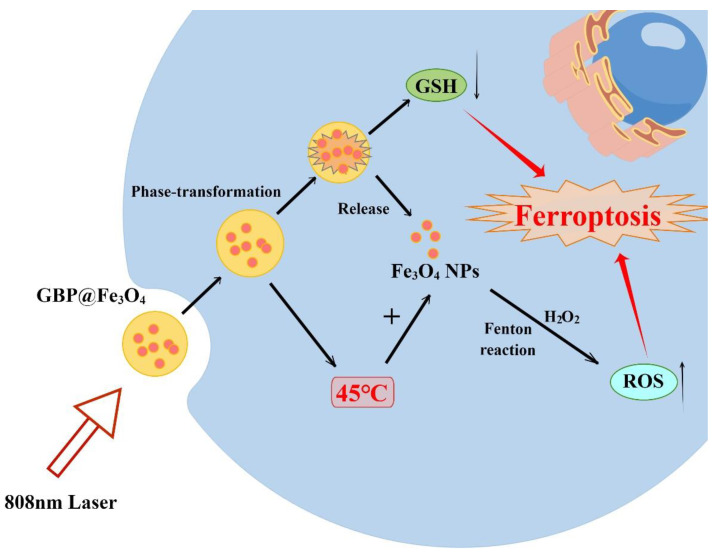
Mechanism of GBP@Fe_3_O_4_-induced ferroptosis. By Figdraw.

Peptide-modified and 1H-perfluoropentane (1H-PFP)-coated Fe_3_O_4_-NPs (GBP@Fe_3_O_4_) have been rationally designed by researchers to propose a hypothesis of heat-triggered tumor-specific ferroptosis (Figure 8) [125]. When a GBP@Fe_3_O_4_ complex is irradiated by an 808 nm laser, the phase transition of 1H-PFP could be triggered by localized heat (45 °C), which leads to an abrupt release of Fe_3_O_4_-NPs in situ, and then generates intense ROS through the Fenton reaction in the TME. Oxidative damage, antioxidant inhibitory response, and the unique reprogramming of lipid metabolism during heat stress are amplified to induce tumor ferroptosis and achieve sufficient antitumor effects. Biocompatible Fe_3_O_4_-NPs act as a supplier of iron ions and can not only promote ROS production, but also participate in iron metabolism associated with ferroptosis [126]. Moderate heat stress has no significant effect on cellular ROS production, but it can significantly amplify Fe_3_O_4_-NP-induced oxidative stress and induce apoptosis in cancer cells [125]. Therefore, moderate heat stress acts synergistically with Fe_3_O_4_-NPs to weaken the self-defense response of tumors and affect their metabolism, thus regulating their mode of death.

A plate-like Bi_2_Se_3_-Fe_3_O_4_/Au (BFA) nanoplatform was designed for clinical diagnosis and therapy [127], which can increase the rate of the Fenton reaction to enhance ferroptosis therapy with active–passive targeting. This platform benefits from the internal synergistic effects of Fe_3_O_4_-NPs and Au-NPs, as well as external near-infrared light (NIR)-mediated hyperthermia, and could promote hydroxyl radical (·OH) production to enhance intracellular oxidative stress and further induce ferroptosis by inactivating GPX4. At the same time, BFA-NPs can be used as an effective diagnostic agent for photoacoustic (PA), magnetic resonance (MR), and X-ray imaging to guide the synergistic treatment of photothermal-ferroptosis. CdSe/Fe_3_O_4_ nanoplatforms coated with multi-mode polymers had both optical and magnetic functions [128,129]. They co-wrapped with CdSe quantum dots and Fe_3_O_4_-NPs, and it was demonstrated for the first time that they may have biomedical applications based on two-photon and magnetothermal therapies such as bioimaging and anticancer therapy. However, co-wrapping limits CdSe/Fe_3_O_4_ nanoplatforms inducing iron-dependent and LPO-mediated ferroptosis. Chen et al. [106] confirmed the synergistic effect of hyperthermia and Fe_3_O_4_-NPs from another aspect. They designed an ADM/Fe_3_O_4_-MS complex to enhance their antitumor effects by activating ferroptosis under microwave heating.

Extensive studies have shown that thermotherapy is an effective sensitizer of tumor chemoresistancy, which can greatly improve the effectiveness of chemotherapy [130] and enhance the antitumor effect of chemotherapeutic agents [131]. Therefore, the introduction of hyperthermia into the chemoembolization system could significantly improve the efficacy of transcatheter arterial chemoembolization (TACE). In conclusion, moderate heat, rather than a heat source, is essential for the induction of ferroptosis in combination therapy [125].

### 4.3. Promotion of Sonodynamic Therapy (SDT)

Recently, researchers have proposed a combination therapy strategy for glioma based on a non-invasive blood-brain barrier (BBB) opening, biomimetic sound-therapy-system-mediated SDT, and ferroptosis [132]. In this study, multifunctional homologous tumor-targeting biomimetic nanoparticles (PIOC@CM-NPs) coated with Fe_3_O_4_-NPs and Ce6 were constructed as a nanoultrasound sensitizer, which combined the biomimetic nanoacoustic-sensitizer-mediated SDT with ferroptosis to achieve the effect of synergistic treatment of glioma. It demonstrated that the glioma C6 cell membrane on the surface of nanoparticles allowed selective accumulation of nanosensitized agents in tumors by homologous targeting in vitro. After effective internalization in C6 cells, PIOC@CM-NPs can significantly improve ROS levels and deplete GSH upon ultrasound irradiation, resulting in a loss of GPX4 activity, thereby promoting SDT and ferroptosis to kill glioma C6 cells.

## 5. Conclusions and Perspectives

Ferroptosis, a new iron-dependent programmed death discovered in recent years, has received increasing attention as a unique mechanism of occurrence and resistance, unlike apoptosis, cell necrosis, and cell autophagy. The activation of ferroptosis is widely recognized as a new target for drug discovery, and an increasing number of small molecule compounds have been identified and characterized to induce ferroptosis directly or indirectly by targeting iron metabolism and LPO. However, there are relatively few reports on the relationship between nanomaterials and cell ferroptosis, especially Fe_3_O_4_-NPs, which are rich in Fe^2+^ and Fe^3+^ and have unique advantages in inducing ferroptosis; however, the specific role and influencing mechanism still requires further research. Although there are many in vitro assays available to detect ferroptosis, such as by measuring cell viability, iron content, and ROS levels, confirming the presence of ferroptosis in vivo is more difficult. Ferroptosis is a double-edged sword, and we need to fully investigate the potential toxicities of inducers or inhibitors of key proteins and pathways of ferroptosis to ensure tumor-specific triggering of the Fenton response and avoid off-target toxicity to normal tissues causing carcinogenesis or other diseases [133,134].
